# Modelling the Impact of Reporting Rates on Outbreak Detection With Implications for Managing Emergency Animal Diseases

**DOI:** 10.1155/tbed/8236134

**Published:** 2026-06-11

**Authors:** Isobel R. Abell, Thao P. Le, Jennifer A. Flegg, Christopher M. Baker

**Affiliations:** ^1^ School of Mathematics and Statistics, University of Melbourne, Victoria, Australia, unimelb.edu.au

**Keywords:** agricultural disease management, mathematical modelling

## Abstract

The detection and surveillance of emergency animal disease outbreaks play a crucial role in their management. However, monitoring such diseases can often rely on farmers self‐reporting animal infection. If there are disincentives for farmers to report disease, this can lead to delayed reporting, which could negatively impact outbreak management outcomes. To understand the range of impacts farmer reporting rates could have on the spread and management of outbreaks, we model the spread of animal disease using an agent‐based model. We investigate how varying the reporting rate for infected properties can impact firstly the total number of properties depopulated for various management strategies and secondly which strategy is optimal. Our model considers disease transmission occurring both within a property, through animal‐to‐animal transmission and between properties, through fomite dispersal and random movement of animals. Using this model, we compare the number of properties depopulated under four strategies: depopulating infected properties and animal movement restrictions combined with (1) ring culling, (2) ring testing, (3) ring vaccination (with a perfect vaccine) and (4) ring vaccination (with an imperfect vaccine). Given our assumptions and model structure, we find that outbreaks spread in clusters around an initially infected property, with additional clusters being seeded early by the random movement of animals between properties. We simulate the impact of reporting rates on a range of outbreak indicators, and we find it has the biggest impact on the time to detection. This time decreases as we increase the reporting rate, but with diminishing returns. We find the timing of outbreak detection impacts the total properties depopulated under all management strategies similarly, as outbreaks detected earlier ultimately lead to smaller outbreaks. As such, we find that varying the reporting rate had minimal impact on which strategy was optimal for a given simulation. Our modelling demonstrates how human behaviour, such as reporting rates, can impact the outcomes from managing emergency animal disease outbreaks. While exact behaviour cannot be predicted for future outbreaks, we can prepare for the next outbreak of emergency animal disease by investigating which management strategies can be robust to a variety of human behaviours.

## 1. Introduction

Early detection is crucial for effectively managing invasive species, human disease and animal disease outbreaks, but this often relies on human behaviours such as reporting disease or infestations. For animal diseases, early outbreak detection can help minimise a range of impacts, for example, reducing negative impacts to animal welfare while reducing the economic cost of both management and outbreak outcomes [1]. However, to design effective surveillance and management strategies, decision makers need to ensure the benefits of a management strategy outweigh its negative impacts [2–4]. The detection and management of animal disease outbreaks also relies on collaboration and shared trust between decision makers and farmers [5]. Decision makers must therefore consider the impact of farmer incentives and behaviour to effectively manage emergency animal disease outbreaks.

While important to consider when managing animal diseases, the exact impact of human behaviour can be hard to predict. Policies are often designed to incentivise farmers to comply with a range of management strategies such as animal movement restrictions, reporting infection and voluntary vaccination of animals [6]. However, there are also factors that limit farmer compliance with these strategies, such as staffing resources available to implement a strategy (e.g. veterinarians), lack of knowledge of clinical signs of infection and the cost to farmers of implementing a strategy (e.g. cost of a vaccine) [7–10]. Interrogating which behaviours may occur in the event of an outbreak is important as non‐compliance with management directives can undermine the overall impact of the strategy [11]. That is, the theoretically optimal strategy may not be realistically optimal in the face of human behaviour.

Mathematical modelling is an invaluable tool for designing and assessing management strategies for animal disease outbreaks. Among other evidence, mathematical modelling can help evaluate the success and impact of different outbreak management strategies [12, 13]. Considering foot‐and‐mouth disease as an example, modelling can help simulate outbreak scenarios [1, 14], determine effective and reasonable management strategies [15–17] and evaluate the economic impact of an outbreak [3, 18]. Studies in this area typically fall into two categories: modelling a specific disease or outbreak or developing general management theory. This paper falls into the latter category. When considering specific diseases and outbreaks, modelling studies can require large amounts of data and are often limited by the quality of the data available [19]. General mathematical studies of outbreak management fill this gap where data is not available, contributing to outbreak preparedness exercises. Furthermore, mathematical modelling can help infer the impact of human behaviour on specific outbreaks of diseases and invasive species. For example, how human behaviours influence the spread of disease and change over the course of an outbreak have been studied in the context of the COVID‐19 pandemic [20, 21]. In invasive species management, notifications of fireant incursions by citizen scientists can impact how an outbreak is managed and subsequently progresses [22]. When human behaviour is known, mathematical modelling can help determine its impact to design effective outbreak management strategies that can reasonably be implemented.

Understanding the impact of human behaviour on outbreak management is crucial to developing robust and effective policies. Outbreak management strategies incorporating culling, vaccination and testing of animals are often employed to help reduce the spread of animal disease outbreaks. Strategies such as ring culling, that is, culling animals on properties (depopulation) surrounding an infected property, are often employed to quickly contain disease spread [23]. Vaccinating at‐risk properties (reactive vaccination) can also be implemented to reduce spread, but its impact can depend on factors such as when vaccines become available, vaccine efficacy, and whether vaccinated animals may still be able to carry disease [14, 24]. Any management strategy has logistic challenges, the biggest of which is often managing limited staffing resources between depopulating, vaccinating or testing premises [2]. Understanding which real‐world factors, such as logistics, economics and social licence, have the biggest impact on management outcomes is crucial for successful outbreak management policies. Mathematical modelling allows us to pull policy levers in a risk‐free environment, understanding how varying behaviours flow on to management outcomes to guide policy design.

In an emergency outbreak scenario, decision makers may face the challenge of designing management strategies without knowing exactly how people will react to them. To help this problem, we can gather information outside of an emergency outbreak scenario in an attempt to predict human behaviour during an outbreak. This can be done by surveying people about their behaviour during a past outbreak or how they would behave in a potential outbreak (e.g. [6–11]). Hill et al. extend this idea by incorporating known human behaviours into mathematical models of animal disease spread [25, 26]. However, collecting data to understand human behaviour is a monumental task. Rather than quantifying human behaviour through collecting data, we can reframe the problem by considering the impacts of a range of behaviours. When we do not know how people will react, by investigating the range of potential behaviours we can assess the range of effects on disease spread and outbreak management.

In our study, we use mathematical modelling to explore the range of impacts that varying farmer reporting rates can have on the spread and management of animal diseases. We define an agent‐based model of animal disease spread both within properties and between neighbouring properties. Considering parameters relevant to an outbreak of foot‐and‐mouth disease in dairy cattle in South Gippsland, Victoria, Australia, we focus on four management strategies: depopulating infected properties and movement restrictions in combination with (1) ring culling, (2) ring testing, (3) ring vaccination (perfect) and (4) ring vaccination (imperfect), respectively. For each strategy, we vary the farmer reporting rate and measure its impact on the total number of properties that are depopulated. Through comparing the number of depopulated properties for each strategy, we explore how the optimal strategy in each scenario varies with the reporting rate.

## 2. Methods

Our model considers the spread of an animal disease within and between properties. We do this through developing a stochastic agent‐based model of disease spread on a generated set of properties. This section describes the details and assumptions of the model.

Our modelling focuses on capturing the dynamics of disease spread on and between properties of animals to understand the impact of farmer reporting rates in a generalised setting. As such, exact distances and parameters for specific diseases are not as important to this study as creating a system to simulate disease spread through a network of properties. Through interrogating this simulated system, we can assess how different reporting rates impact the overall disease spread and outbreak management. In this section, we build a generalised model of disease spread, but results will consider parameters relevant to a foot‐and‐mouth outbreak among dairy cattle in South Gippsland, Victoria, Australia.

### 2.1. Property Set Initialisation

Each time we run a simulation of our model, we generate a new set of properties and their locations. For each of the results presented in this paper, we consider properties in a 20 km×20 km area.

To generate the set of properties, we randomly assign each property coordinates and area one by one. In our simulations, we assume that each property has the same area. Each time we assign property coordinates, if one property’s area overlaps with another, we assign new coordinates and continue until there is no overlap. This ensures that properties do not overlap with one another.

### 2.2. Mechanisms of Disease Spread

**Figure 1:**
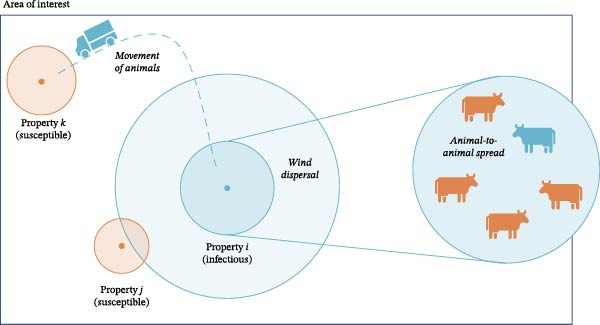
A diagram showing the three disease transmission mechanisms included in our modelling. Here, blue represents infection, that is, property *i* is infected, and the blue animal on property *i* is infectious. Orange represents susceptibility, that is, properties *j* and *k* are susceptible and the orange animals on property *i* are susceptible.

In our model, we consider three mechanisms of disease spread: animal‐to‐animal, wind dispersal of fomites and movement of animals, as shown in Figure 1. Animal‐to‐animal transmission and transmission by wind dispersal of fomites can contribute to localised spread of diseases such as foot‐and‐mouth disease, avian influenza and classical swine fever [27–29]. The movement of infected animals can also extend an outbreak of such diseases [30].

**Figure 2:**
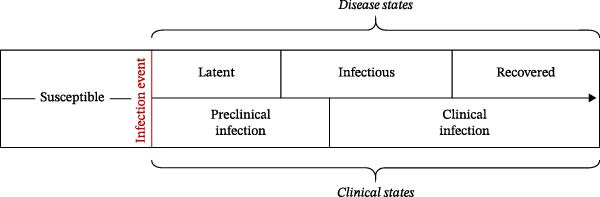
A summary of the disease and clinical states of infection for animal‐to‐animal transmission for animals considered in our modelling. The disease states on the top row relate to an animal’s susceptibility and ability to transmit disease. The clinical states on the bottom row relate to the visibility of infection to farmers.

We model the disease spread using an SEIR model framework. The timeline of disease and clinical states considered in our modelling is shown in Figure 2. We assume that animals start ‘susceptible’ and, upon infection, become ‘exposed’. Once exposed, animals will transition to ‘infectious’ and, from there, to ‘recovered’. As we are ultimately interested in the dynamics of farmers reporting infection, we also consider the clinical signs of infection in animals. Upon infection, animals are designated as ‘preclinical’, where they do not exhibit any symptoms of disease. From here, they transition to a ‘clinical’ disease, where they will exhibit symptoms of disease. In our modelling, we assume that only animals with clinical infection can be detected (and potentially reported) by farmers.

Our modelling considers discrete time, that is, disease progression, spread and reporting occur daily. To model disease spread, we define the force of infection and use this to calculate an animal’s daily probability of being infected. We assume all animals progress through disease at the same rate, and this is incremented daily in our modelling.

#### 2.2.1. Animal‐to‐Animal Transmission

We define animal‐to‐animal transmission as for a SEIR model with density‐dependent transmission. That is, we define the animal‐to‐animal force of infection on property *i*, *λ*_*i*, *A*_(*t*), as

(1) λi,At=βAIit,

where

• *β*_*A*_ is the transmission rate, and
• *I*_*i*_(*t*) is the number of infectious animals on property *i* on day *t*.

Another important parameter we define is *R*_0_: the average number of infections arising from one infected animal in a fully susceptible population. When specifying values for parameters in our modelling, we specify a value for *R*_0_ and fit our model to this to find *β*_*A*_, rather than specifying *β*_*A*_ itself. Details on this calibration process can be found in Appendix A.

#### 2.2.2. Transmission via Wind Dispersal of Infectious Material

To define the force of infection from the dispersal of fomites on property *i*, *λ*_*F*,*i*_, we first consider a disc around a property over which fomites can be dispersed. We define this disc by the fomite dispersal radius *r*_*F*_.

Given this disc around an infected property, we make the following assumptions:

1. The force of infection from wind dispersal is proportional to the cumulative infections on a property, *C*_*i*_(*t*). That is

(2) λi,Ft∼Ci.

2. The infectious material is distributed equally across the area of the fomite dispersal disc.
3. Only properties whose area overlaps the fomite dispersal disc of an infected property can be infected by it. The contribution to a property’s force of infection from an infected neighbour is defined as proportional to the proportion of the fomite dispersal disc of the neighbouring property *j* that overlaps with the considered property *i* (see Figure 1 for a visualisation of this overlap). Defining this overlap as *A*_*i*,*j*,*F*_ and the fomite dispersal disc of property *j* as *A*_*j*,*F*_,

(3) λi,Ft∼Ai,j,FAj,F.

4. The force of infection is additionally proportional to the distance between the centre of an infected property and the boundary of its neighbour. That is, the further away neighbouring properties are, the less impact neighbouring infections have. As properties cannot be impacted outside the fomite dispersal radius *r*_*F*_, we assume this proportionality is linear with the following form:

(4) λi,Ft∼1−di,jrF,

where *d*_*i*,*j*_ is the distance from the boundary of property *i* to the centre of property *j*.

Combining each of these assumptions, we define the force of infection acting on a property *i* due to the dispersal of fomites (*λ*_*i*,*F*_(*t*)) as

(5) λi,Ft=βF∑j∈PCjtAi,j,FAj,F1−di,jrF,

where given a property *i*:

• *β*_*F*_ is the transmission rate due to wind‐dispersed fomites,
• *P* is the set of properties within the wind radius of property *i* (including property *i* itself). Considering a property *j* ∈ *P*:
• *C*_*j*_(*t*) is the number of cumulative infections on property *i*,
• *A*_*j*,*F*_ is the area of the wind dispersal centred on property *j*,
• *A*_*i*,*j*,*F*_ is the area of intersection between the area of property *i* and the wind dispersal disc centred on property *i*. *A*_*i*,*i*,*F*_ is, by definition, the area of property *i*,
• *d*_*i*,*j*_ is the minimum distance between the boundary of property *i* and the centre of property *i* (we define *d*_*i*,*i*_  = 0) and
• *r*_*F*_ is the radius of the wind dispersal disc.

In our model, we have incorporated a simple linear transmission kernel to capture how transmission varies with distance. Specifically, we assume that transmission decays linearly to the radius of the wind dispersal disc, after which we assume there is no transmission:

Transmission kerneldi,j=1−di,jrF,0<di,j≤rF,0,di,j>rF.

There are many other forms that can be used when modelling various diseases with spatial transmission (e.g. [23, 31]). Often, spatial kernels are fit to data from a particular outbreak, making them highly specific, hard to interpret and not transferrable to other outbreaks [32]. We use a linear kernel parameterised by the minimum distance between properties and the fomite dispersal radius for simplicity and interpretability within the context of our modelling.

#### 2.2.3. Force of Infection

Combining the animal‐to‐animal force of infection (Equation 1) and the force of infection from the wind dispersal of fomites (Equation 5), we calculate the total force of infection on property *i*, *λ*_*i*_(*t*), as

λit=λi,At+λi,Ft,

⇒λit=βAIit+βF∑j∈PCjtAi,j,FAj,F1−di,jrF,

with parameters as defined in Sections 2.2.1 and 2.2.2.

We assume that the daily probability of infection for a susceptible animal follows decaying exponential dynamics (as assumed for SEIR‐type deterministic models but explicitly implemented here). We therefore define the probability of infection for a susceptible animal on property *i* on day *t*, Pr(infection)_*i*_(*t*), as

Prinfectionit=exp−λit,

Prinfectionit=exp−βAIit+βF∑j∈PCjtAi,j,FAj,F1−di,jrF.

#### 2.2.4. Movement of Animals

Animals are regularly moved between properties, leading to transmission due to the movement of infected animals. However, animal movement data between farms is not publicly available in Australia. As such, our model incorporates the random movement of animals determined by the frequency of movement (*f*), probability of movement (*p*_*m*_) and number of animals moved (*n*_*m*_). We define the movement frequency by how often animals are moved from a property, for example, once every week, once every month and others. Given the frequency *f*, each property in our model is assigned a random starting movement date within the movement time period to ensure movements are not synchronised across the network of properties. Furthermore, each property has a probability *p*_*m*_ that animals will be moved from that property. As such, if it is a movement day and animals are moved, *n*_*m*_ animals (where *n*_*m*_ is between 0 and the total number of animals on the property) are chosen randomly from the property and moved to another property in the network. We assume every property can reach every other property in the network and animals move to a random property.

Movement restrictions, whether within a determined radius of an infected property or a standstill across a wider area, are often the first management strategy for emergency animal disease outbreaks (e.g. Animal Health [33–35]). In our model, we assume that there is a movement standstill across all properties following the first report of an infected property, reflecting local movement restrictions.

### 2.3. Disease Management and Surveillance

#### 2.3.1. Ring Actions

When a property is designated as infected, a chosen management strategy is initiated. In our modelling, we consider four separate management actions: depopulating infected premises and movement restrictions combined with (1) ring culling, (2) ring testing, (3) ring vaccination (perfect) and (4) ring vaccination (imperfect), respectively. Given a radius of ring management, we implement ring actions by applying management to properties within the radius of an infected property. To reflect logistic constraints on implementing strategies (e.g. sending veterinarians out to an infected property to cull or vaccinate animals), we assume that there is a delay between an action being decided upon and the action taking place.

In our results, we consider each management strategy independently. If ring culling is implemented, neighbouring properties within the ring radius will be depopulated after a specified delay. If ring vaccination (perfect or imperfect) is implemented, these properties will be vaccinated after the specified delay. If ring testing is implemented, neighbouring properties will be tested after the specified delay. If these properties test positive for infection, they will be depopulated after another delay. We assume 100% specificity and 100% sensitivity for the testing.

#### 2.3.2. Reporting Property Infection

In our model, there is a daily probability that an infected property will report their infection status. If a farmer reports their infection status, the property is depopulated and the considered management strategy is initiated.

We define *r* as the probability a farmer will report their property as infected given all animals are infected. We assume the daily reporting probability for a given property is dependent only on *r* and the proportion of infected animals at that property. We can therefore calculate the probability that property *i* reports their infection status on day *t* (*P*_*R*,*i*_(*t*)) as

PR,it=rIitNit,

where *I*_*i*_(*t*) is the number of infected animals on property *i* on day *t* and *N*_*i*_(*t*) is the total number of animals on property *i* on day *t*.

For the simulations presented in our results, we vary *r* between values of 0.1 and 1. As self‐reporting is the only way to initially detect an outbreak in our model, if *r* = 0, the outbreak will never be detected. As such, we will only consider *r* > 0 in our modelling.

### 2.4. Modelling Details

#### 2.4.1. Model Parameters

While we have defined our model generally for a range of animal diseases, we select a specific context to parameterise the results for this paper. We consider an outbreak of foot‐and‐mouth disease in dairy farms in South Gippsland, Victoria, Australia. Gippsland is home to around 1000 of Australia’s dairy farms, and 40% of these farms are found in South Gippsland, in an area of around 3300 km^2^. These dairy farms have an average property radius of 1 km and an average of 350 cattle (agriculture [36]).

In our model, we consider a 20 km×20 km region of South Gippsland, containing 40 dairy farms with 350 cattle on each. We assume that these properties are randomly spread throughout the region. In addition, we choose disease parameters relevant to foot‐and‐mouth disease. Table 1 defines the parameters used in our modelling.

**Table 1:** Parameters used in our modelling, their definitions, values and supporting references.

Name	Definition	Value	Source	Notes
Transmission parameters				
**R**_0_	The average number of infections arising from one infected individual in a fully susceptible population.	2	[37–40]	*R*_0_ is not explicitly specified in our model, but instead used to calibrate *β*_**A**_ and *β*_**W**_.
*β*_**A**_	The rate at which a susceptible individual is infected on contact with an infected individual.	Calibrated to **R**_0_	N A	—
*β*_**F**_	The rate of infection on contact with dispersed fomites.	0.5*β*_**A**_	N A	We arbitrarily assume *β*_**F**_ is 50% of *β*_**A**_
Infectious period	The average length of time an individual spends infectious	6 days	[37, 40]	—
Latency period	The average length of time from when an individual first becomes infected and when they become infectious.	2 days	[37]	—
Incubation period	The average length of time from when an individual first becomes infected and when they show symptoms.	3 days	[35, 38]	—

Property parameters				
**N**_**i**_	The number of animals on property *i*.	350	[36]	The average size of a dairy cattle farm in South Gippsland, Victoria, Australia.
Property radius	The radius of each property in the network.	1 km	[36]	—

Property set parameters				
Grid size	Size of the coordinate grid on which each network is generated.	20 km × 20 km	N A	An appropriate size *n* = 40 dairy cattle properties in South Gippsland, Victoria, Australia.
**n**	The number of properties in each generated network.	40	N A	Chosen for this study.

Ring action parameters				
**r**_**F**_	The fomite dispersal radius, the maximum distance for infection between two properties.	2 km	N A	We assume this is larger than the radius of one property to model infection between properties.
**r**_**A**_	The ring action radius, the distance within which management occurs around an infected property.	3 km	[35]	We assume a restricted area radius as specified for foot‐and‐mouth disease.
**r**	The probability a farmer reports their property as infected given all animals are infected.	0.5	N A	We vary *r* between 0.1 and 1, but use a default of 0.5 unless otherwise stated.
Clinical reporting threshold	The proportion of infections over which farmers can see signs of infection and possibly report their property as infected.	0.05	[41]	—
**v**	The reduction of susceptibility due to vaccination. *v* = 1 indicates no reduction, *v* = 0 indicates full protection from infection.	0 (perfect), 0.2 (imperfect)	N A	—
Ring action delay	The time from which a ring action is designated to occur on a property, and when it actually occurs.	5 days	N A	Chosen arbitrarily for our study.

Movement parameters				
**f**	Movement frequency, how often random movement of animals between properties occurs.	Once every 7 days	N A	Due to the lack of publicly available data, movement parameters have been chosen arbitrarily for this study.
**p**_**m**_	The probability animals are moved from a given property.	0.4	N A	—
**n**_**m**_	The number of animals moved from a property.	20% of the animals on a property	N A	—

Simulation parameters				
Simulations	The number of simulation instances generated for our results.	1000	N A	Chosen for to generate stable results within a reasonable time frame.

*Note:* For the results in this paper, we consider parameters relevant for a foot‐and‐mouth disease outbreak in dairy cattle in South Gippsland, Victoria, Australia. Where there are no references (N/A), it indicates these parameters have been chosen for this study.

#### 2.4.2. Simulation Details

The simulation of our model proceeds as follows:

1. Set seed: In order to determine which is the optimal management strategy for a given scenario, we first set a random seed. This ensures that we compare like‐to‐like when determining the optimal strategy for a given scenario.
2. Initialise properties: We initialise the properties and their coordinates in our 20 km × 20 km space.
3. Seed an outbreak: We seed an outbreak by designating a random property infected. We then designate 5 animals on that property as infectious.
4. Choosing a management action: Each management action (depopulating infected properties combined with ring culling, testing or vaccination) is implemented one by one.
5. Simulate a single outbreak: Our model runs until there are no more infections on any properties and there are no delays in place, that is, no property is waiting to be depopulated/vaccinated/tested. While there may still be a non‐zero force of infection from fomite dispersal (as this depends on cumulative infections), we assume that this could potentially seed a *new* wave of infection. That is, at this point, we consider one epidemic wave to have finished and hence finish our simulation.

The summary statistics presented in our results consider 1000 simulation instances and default parameters, as defined in Table 1. For details on the implementation of our model and access to the modelling code, see https://github.com/iabell/modelling-reporting-rates.

## 3. Results

In this section, we demonstrate the overall dynamics of disease spread in our model as well as the impact of varying the reporting rate on the outcomes of management strategies. We start by describing the general model dynamics as seen in each simulation instance. We then investigate how the timing of outbreak detection varies with the reporting rate. Finally, we consider how reporting rates impact how many properties are depopulated under each management strategy and how these values compare between strategies.

We define our management objective as minimising the number of properties depopulated during an outbreak and consider strategies as described in Section 2.3.1. Results for additional management objectives are shown in Appendix A.

### 3.1. Model Dynamics

**Figure 3:**
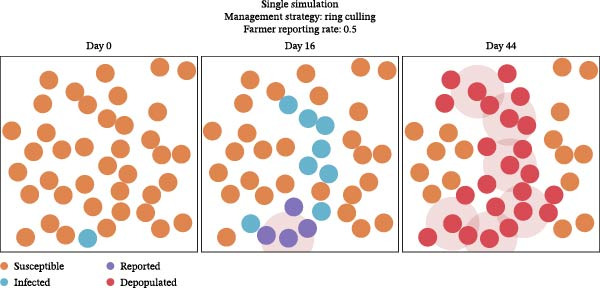
A single simulation instance of our model showing three key time points: the start of the outbreak, the day the outbreak is first reported and the end of the outbreak. The first panel shows Day 0 of the simulation, highlighting the initially seeded property in the network. The second panel shows Day 16 of the simulation, highlighting the clusters seeded by random movement of animals by the time a property first reports infection. After this time point there is no random movement of animals between properties. The third panel shows the final day of the simulation (Day 44), highlighting the total spread of disease throughout the network. The properties in each figure are coloured according to their disease and management status as follows: orange – susceptible, blue – infected, purple – reported, red – depopulated. The coloured disc around a property indicates the management area around a reported property. Properties within this disc are also shown as reported, as they will be depopulated under the ring culling strategy.

Figure 3 shows a single simulation instance of our model, demonstrating how the three mechanisms of disease spread (as defined in Section 2.2) determine how an outbreak progresses. The simulation begins with seeded infectious animals on a random property (Day 0 in Figure 3). As the number of infectious animals on this property increases, there is an increased chance of the disease spreading to neighbouring properties. So, we see infected properties clustering around the initially infected property. New clusters are seeded by the random movement of infectious animals from infected properties to susceptible properties.

By the time a property first reports infection (Day 16 in Figure 3), we see two distinct clusters of infected properties. Once the first infection report occurs, movement restrictions are initiated and no further random movement occurs. The remainder of disease spread between properties is due only to wind dispersal of fomites. We therefore see disease spread from the clusters of infection, mitigated by chosen management actions (e.g. ring culling) until there are no more infections on any property (Day 44 in Figure 3).

Interrogating this simulation provides a baseline for understanding the rest of our results. The dynamics described here influence the following results, which are analysed using summary statistics from multiple simulations. Varying parameters such as those that govern movement and disease characteristics will impact the dynamics of an outbreak, but we hold these parameters constant over the rest of our results, varying only the reporting rate.

### 3.2. Timing of Outbreak Detection

**Figure 4:**
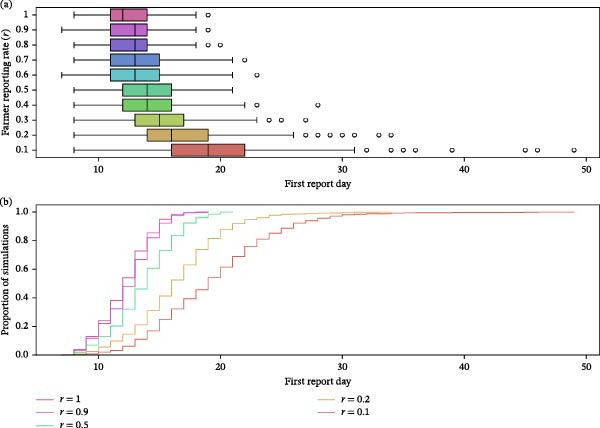
The days on which the first property reports infection as we vary the farmer reporting rate (*r*). Figure (a) shows the distribution of first report days for each *r* value. Figure (b) shows the empirical cumulative distributions for select *r* values. We consider 1000 simulations for each *r* value, with default parameters as described in Table 1. Each simulation considers a ring culling strategy, although the strategy chosen will not impact the first report day. Simulations where the epidemic dies out before being reported were excluded (32 simulations).

In our model, the time at which an outbreak is first detected is independent of the management strategy considered and instead solely dependent on individual properties reporting their infection. Figure 4 demonstrates how the day an outbreak is first reported varies with the farmer reporting rate *r*.

As we increase the reporting rate *r*, we see an increase in simulations where properties report earlier, though with diminishing returns. Furthermore, we see the distribution of first report days widen as we decrease *r*. The left tail of the distribution is consistent across all values of *r* due to the clinical reporting threshold. That is, the number of animals with clinical infection needs to exceed the clinical reporting threshold (5%) before it becomes possible to report infection in our model. This puts a lower bound on the first report day, and as the disease parameters are held constant for all simulations, we therefore see a similar lower bound for all *r* values. However, the upper bound for first report days increases as we decrease *r*. When a property is fully infected, the daily probability a property reports is equal to *r*. As such, with high reporting rates (*r* > 0.5) a property is more likely than not to report when all animals have been infected (the daily probability is between 0.5 and 1). There is, therefore, little difference in the first report day for these high rates, as can be seen for the *r* > 0.5 distributions in Figure 4. For lower reporting rates, particularly *r* =  0.1 and *r* = 0.2, even when a property has all animals infected, there is a small probability the property reports. As such, we see later first report days appearing in the simulations for low *r* values, widening the distributions.

### 3.3. Comparing Management Strategies

**Figure 5:**
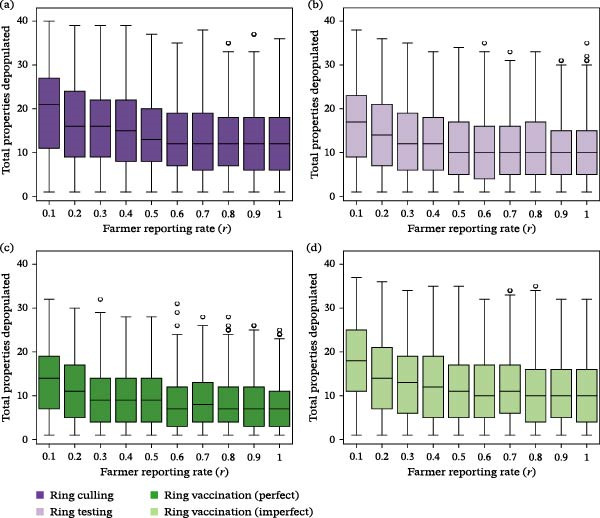
The total number of properties depopulated under strategies of depopulating infected properties and movement restrictions combined with (a) ring culling, (b) ring testing, (c) ring vaccination (perfect) and (d) ring vaccination (imperfect) strategies as we vary the reporting rate (r). 1000 simulations are generated per *r* value for each strategy. Simulations where the epidemic dies out (10 total) are removed from the results.

Figure 5 depicts how farmer reporting rates impact the number of properties depopulated during an outbreak under various management strategies.

As we increase the reporting rate *r*, we see a decreasing trend in the inter‐quartile range for total properties depopulated across all management strategies. In Figure 4, we see diminishing returns on the first report day and narrowing distributions as we increase the reporting rate. We also see diminishing returns on the median total depopulated properties but instead see widening distributions as we increase *r* for all management strategies. For the lower *r* values, we see more simulations where high numbers of properties are depopulated across all strategies. For lower reporting rates, delayed reporting of infection leads to greater outbreaks and hence more properties being depopulated. However, even for higher reporting rates, there are still some simulations with extensive spread despite potentially earlier outbreak notification. Overall, increasing the reporting rate decreases the median properties depopulated for all management strategies; however, the stochasticity inherent in our modelling means we see simulations with extensive disease spread and depopulation even for high reporting rates.

### 3.4. Optimality when Varying Reporting Rates

**Figure 6:**
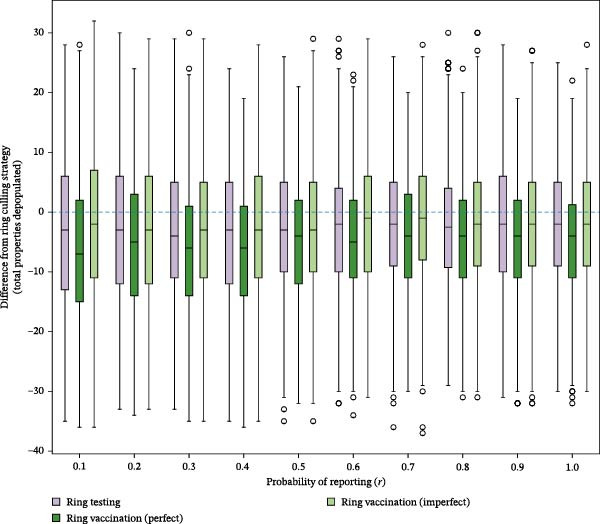
The difference in depopulated properties for each simulation is from a ring culling strategy for ring testing and ring vaccination (perfect and imperfect) strategies. A positive value means the considered strategy is worse than ring culling, that, it results in more depopulated properties. A negative value means the considered strategy is better than ring culling, that, it results in fewer depopulated properties. 1000 simulations were generated per *r* value for each strategy. Simulations where the epidemic dies out before being detected were discarded (32 simulations).

**Figure 7:**
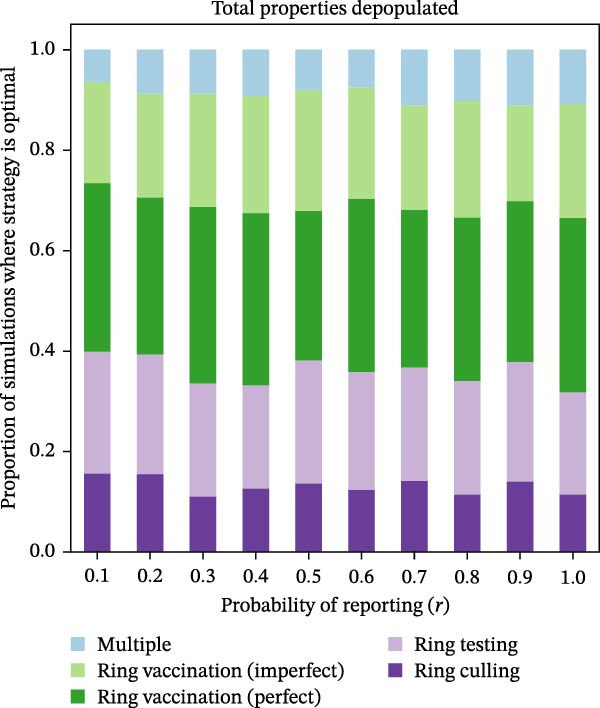
The proportion of simulations for which ring culling, ring testing and ring vaccination strategies are the optimal strategies to minimise total properties depopulated. 1000 simulations were generated for each *r* value and simulations where the epidemic dies out before being detected were discarded. ‘Multiple’ indicates simulations where there was more than one optimal strategy.

Given the total properties depopulated for each management strategy, as shown in Figure 5, we now consider which strategy minimises the total properties depopulated as we vary the reporting rate. Figure 6 shows the difference in depopulated properties under ring testing, ring vaccination (perfect) and ring vaccination (imperfect) strategies compared to those under a ring culling strategy. This figure presents the same data as Figure 5, comparing the outcomes of each strategy with one another. Figure 7 shows the proportion of simulations for which each considered strategy is optimal (minimises the number of properties depopulated) across different reporting rates. Table 2 presents the data for Figure 7 alongside the mean, variance and standard deviation across all reporting rates for each management strategy.

**Table 2:** The proportion of simulations where one strategy is optimal for varying reporting rates (as shown in Figure 7).

Reporting probability	Proportion of simulations where strategy is optimal				
	Ring culling	Ring testing	Ring vaccination (perfect)	Ring vaccination (imperfect)	Multiple
0.1	0.16	0.24	0.34	0.20	0.064
0.2	0.15	0.24	0.31	0.21	0.088
0.3	0.11	0.23	0.35	0.23	0.087
0.4	0.13	0.21	0.34	0.23	0.092
0.5	0.14	0.25	0.30	0.24	0.081
0.6	0.12	0.24	0.35	0.22	0.076
0.7	0.14	0.23	0.31	0.21	0.11
0.8	0.11	0.23	0.33	0.23	0.10
0.9	0.14	0.24	0.32	0.19	0.11
1	0.11	0.20	0.35	0.23	0.11

Mean	0.13	0.23	0.33	0.22	0.09
Variance	0.00027	0.00021	0.00032	0.00025	0.00025
SD	0.017	0.014	0.018	0.016	0.016

*Note:* The mean, variance and standard deviation (SD) for each strategy (across all reporting probabilities) are also given. 1000 simulations were generated for each reporting rate. ‘Multiple’ indicates the proportion of simulations where there was more than one optimal strategy. Simulations where the epidemic died out before being detected have been removed from the data (32 simulations). Results have been rounded to 2 significant figures.

Our results show that while varying the reporting rate *r* impacts the absolute outcome of management strategies, it has a smaller impact on which strategy is optimal. In Figure 6, we see little change in the median difference and inter‐quartile range for each strategy as we vary *r*. As we see in Figure 4 with the first report day, we see the distributions for properties depopulated narrowly as *r* increases. The medians are consistently negative for ring testing, ring vaccination (perfect) and ring vaccination (imperfect), indicating that on average these strategies result in fewer properties depopulated than ring culling. The ranking of strategies becomes clearer in Figure 7, where we find ring vaccination (perfect) is the optimal strategy for a majority of simulations across all *r* values. Indeed, there is little change in the proportion of simulations for which each strategy is optimal as we vary *r*, quantified by the data presented in Table 2. The standard deviations (SDs) for each strategy across reporting rates are very low, varying from a proportion of 0.014 for ring testing to a proportion of 0.018 for ring vaccination (perfect).

As shown in Figure 4, varying the reporting rate has the biggest impact on the time until an outbreak is first reported in our modelling. While this impacts the absolute outcomes of management strategies, our results show that the number of depopulated properties decreases at a similar rate across all management strategies as we increase the reporting rate. It is therefore unsurprising that the proportion of simulations where a given strategy is optimal varies little with the reporting rate. The difference in proportion we see is mostly due to the effectiveness of the strategies themselves, with ring vaccination (perfect) being most often the optimal strategy in our modelling.

In this study, we have focused on the optimal management strategies to minimise the total number of properties depopulated. The figures in Appendix A consider two different objectives: the total length of an outbreak and total resources used (i.e. teams sent to depopulate/vaccinate/test a property). In Table 2, we see that ring culling is least often the optimal strategy when minimising total property depopulation. However, ring culling is most often the optimal strategy when considering minimising both the length of an outbreak and the total resources used, respectively (Appendix A: Figures 1–6). This presents a trade‐off for ring culling in our modelling. Ring culling is a very effective management strategy for quickly suppressing an outbreak and therefore managing the available resources. However, this comes at the cost of further property depopulation, which could be avoided using alternative strategies.

## 4. Discussion

To investigate the impact of a range of farmer reporting behaviours, we develop a mechanistic model of animal disease incorporating disease spread through animal‐to‐animal contact, wind dispersal of fomites and random movement of animals. We consider four management strategies: depopulating infected properties combined with ring culling, ring testing, ring vaccination (perfect) and ring vaccination (imperfect), respectively. We find that the time to detection is greatly impacted by varying the reporting rate. For lower reporting rates, the simulated outbreaks are often detected later, leading to the extensive spread of disease and thus higher numbers of total properties depopulated for all management strategies. However, in our modelling results, increasing the reporting rate has diminishing returns on the number of properties depopulated—if a property has a high proportion of infected animals, they are likely to report this infection regardless of the reporting rate. Furthermore, we find that the number of depopulated properties decreases similarly across all management strategies as we increase the reporting rate. Ring vaccination (perfect) is most frequently the optimal strategy (being optimal in 33% of simulations, averaged across reporting values). However, changing the reporting rate makes very little difference to how often any particular strategy is optimal (e.g. with ring vaccination (perfect) being optimal in 42% to 55% of simulations).

By simulating outbreak dynamics, our modelling helps us understand how reporting behaviour could impact management outcomes in real‐world scenarios. Our modelling presents one scenario of a disease outbreak, and we consider how varying the reporting rate impacts management strategies given this one scenario. In this case, early detection was most important to the outcome of management strategies; varying reporting rates, while impacting total depopulated properties, did not change which management strategy was most often optimal. If this scenario was seen in a real‐world outbreak, our modelling suggests resources would be best spent improving surveillance for disease to detect outbreaks earlier rather than investigating reporting behaviours to determine the optimal management strategy. However, there are many other factors that influence real‐world outbreaks, including human behaviours such as vaccine hesitancy and the illegal movement of animals. In considering reporting rates in our modelling, we assume that the behaviours of farmers are homogeneous and constant throughout the outbreak. However, the implementation of a specific management strategy may impact farmer behaviour, for example, implementing a ring culling strategy may disincentivise farmers from reporting infection. A natural extension for our study would be to vary the reporting rate across properties, through time, and between management strategies. This would allow us to investigate how changing behaviours during an outbreak can impact the outcome of management strategies implemented.

Modelling real‐world outbreaks requires knowing specific disease mechanisms and incorporating data collected from outbreaks to help decision makers optimise for a desired objective. In some cases, exact mechanisms of diseases are not known, and data can struggle to capture important factors such as illegal movement of animals or disease spread by feral or wild animals. However, by constructing a general model of disease spread within a property and between properties, we can capture a large range of dynamics to understand the spread of diseases in various contexts. Applying our modelling to a real‐life outbreak would require incorporating outbreak data and knowledge of specific disease mechanisms. This could include incorporating known networks of animal movement, for example, through adding abattoirs and saleyards to our modelling. Incorporating such movement data would help us understand the transmission networks, informing decision making for a specific outbreak context.

Furthermore, while we consider a single objective, managing outbreaks of emergency animal diseases is often a multi‐objective problem. In this study, we have chosen to analyse our results in the context of minimising the number of properties depopulated during an outbreak (with objectives of length of an outbreak and total resources used presented in Appendix A). There are, of course, many other objectives to consider, both quantitative (e.g. the economic cost of an outbreak) and qualitative (e.g. social impact). However, focusing on certain objectives can limit the management strategies that are available. For example, minimising the economic cost of an outbreak may suggest attempting to first eradicate a disease without vaccination due to potential trade implications of losing a disease status of ‘free’ without vaccination, as is the case for foot‐and‐mouth disease. Realistically, there is unlikely to be one mathematically optimal management strategy that satisfies the many objectives and constraints considered by decision makers. By looking at dynamics that could emerge during the management of an outbreak, our modelling provides decision makers with tools to think about the diverse impacts of implementing management strategies.

While we can learn from past outbreaks, it is unlikely that we will know exactly how people will behave in the event of a new outbreak of animal disease. Given this, we cannot know how human behaviour will impact the outcomes of management strategies, but by modelling varying behaviour we can hypothesise its impact. To prepare for future animal disease outbreaks, we must anticipate human behaviour where possible and, where not, study the impact of a range of potential behaviours. Our work contributes to emergency animal disease preparedness by investigating reporting rates when considering common mechanisms of spread, but data‐driven studies of disease are needed to support real‐time decision making. This work, considered as part of a wider suite of evidence, aids decision makers in designing management strategies robust to a range of potential and unknown behaviours to prepare for the next emergency animal disease outbreak.

## Author Contributions

All authors conceived the ideas, designed the methodology and conducted the analysis. Isobel R. Abell led the writing of the manuscript.

## Funding

Isobel R. Abell is supported by an Australian Government Research Training Program (RTP) Scholarship doi.org/10.82133/C42F‐K220. Thao P. Le acknowledges funding from the Andrew Sisson Support Package. Open access publishing facilitated by The University of Melbourne, as part of the Wiley ‐ The University of Melbourne agreement via the Council of Australasian University Librarians.

## Disclosure

All authors contributed critically to the drafts and gave final approval for publication. This work was produced as part of the HASTE project. Enhancing Models for Rapid Decision‐Support in Emergency Animal Disease Outbreaks (HASTE) is a co‐investment partnership with the Australian Research Data Commons (ARDC) (DOI:10.47486/DC110). The ARDC is enabled by the Australian Government’s National Collaborative Research Infrastructure Strategy (NCRIS).

## Conflicts of Interest

The authors declare no conflicts of interest.

## Supporting Information

Additional supporting information can be found online in the Supporting Information section.

## Supporting information


**Supporting Information** Appendix A provides extra modelling details and results.

## Data Availability

The code used to generate the results for this paper can be found at https://github.com/iabell/modelling-reporting-rates.
