# Supplementary material for: Modelling the Impact of Reporting Rates on Outbreak Detection With Implications for Managing Emergency Animal Diseases
Source: Transbound Emerg Dis. 2026 Jun 11;2026:8236134. doi: 10.1155/tbed/8236134 (PMC13254539; doi:10.1155/tbed/8236134)
Supplement: Supplementary file 1 — Supporting Information Appendix A provides extra modelling details and results. [file TBED-2026-8236134-s001.pdf]

Modelling the impact of reporting rates on outbreak detection with implications for managing emergency animal diseases

Appendix A

Isobel R. Abell, Thao P. Le, Jennifer A. Flegg, Christopher M. Baker

1 R0 calibration

To calibrate β for a specified R0, we use a rejection-sampling algorithm:

1. Sample β_S: we draw a sample parameter (β_S) from a normal distribution with mean $R_0/(\gamma * N)$ (where R_0 is the specified reproductive number, $1/\gamma$ is the average infectious period and N is the average number of animals on a property), and standard deviation 0.01. This mean comes from the definition of R_0 from a deterministic density dependent SIR model.

2. Calculate R_0: we run our agent based model with $\beta = \beta_S$ for 500 simulations instances, seeding a single infection each instance. For each simulation, we calculate the number of subsequent infections arising from a single infection. We calculate R_0 by taking the average of the number of secondary infections across all simulations.

3. Accept/reject: if $R0$ is within the specified tolerance ($\epsilon = 0.1$), accept β_S otherwise we draw another value and repeat.

4. 100 samples: We continue this process until we have 100 β_S values to sample from when running our ABM.

This process gives us 100 β values to sample from when running our model. That is, for each model instance, we randomly choose one of the β values previously calibrated.

2 Alternative management objectives

For the results of our main paper, we show the total number of properties culled for each management strategy and determine the optimal strategy as minimising the objective. However, this is not the only management objective that can be considered by decision makers during an emergency animal disease outbreak. Here we present results for two other example objectives: length of epidemic and resources used. We define resources used by the number of teams sent out to properties to enact a management action, i.e. the total number of properties culled, vaccinated and tested.

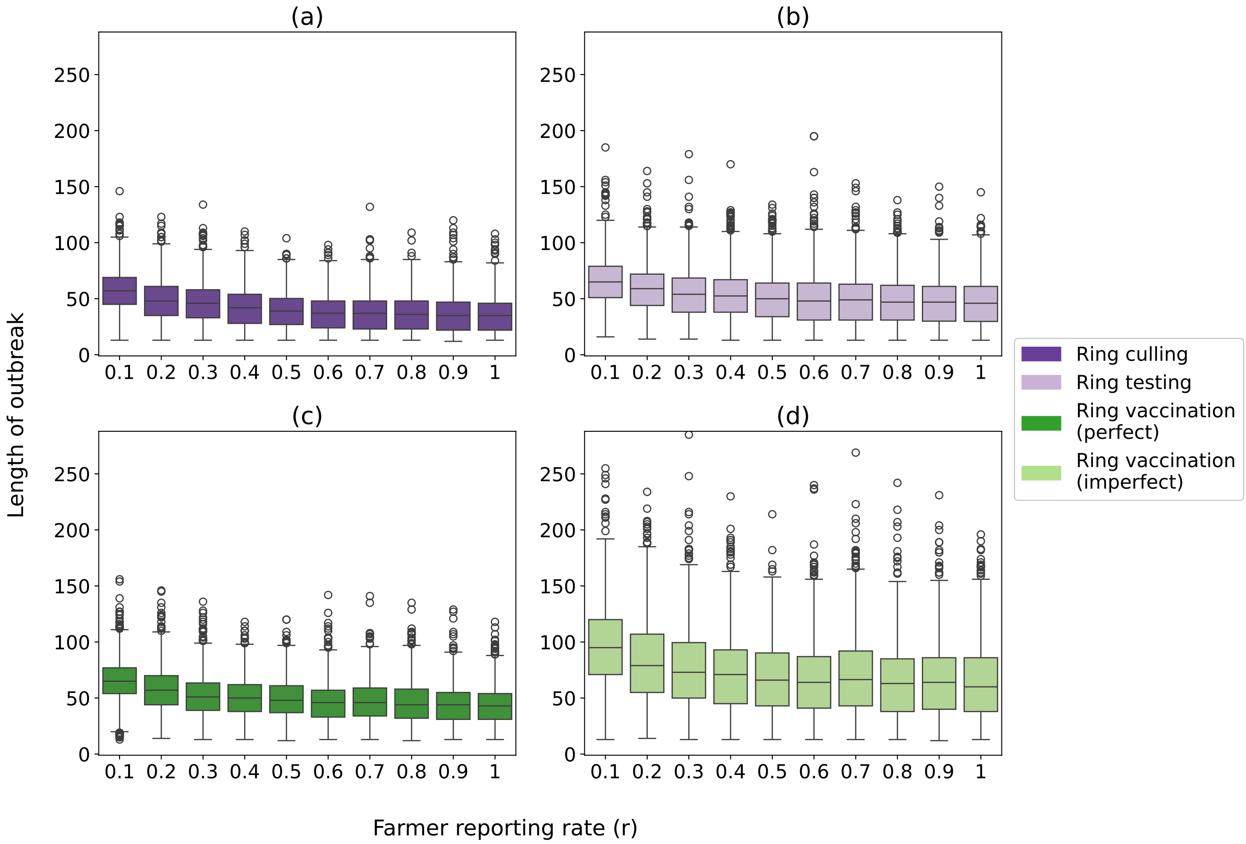

Figure 1: The length of outbreaks under strategies of culling infected properties and movement restrictions combined with (a) ring culling, (b) ring testing, (c) ring vaccination (perfect) and (d) ring vaccination (imperfect) strategies as we vary the reporting rate (r). 1000 simulations are generated per r value for each strategy. Simulations where epidemic dies out (32 total) are removed from the results.

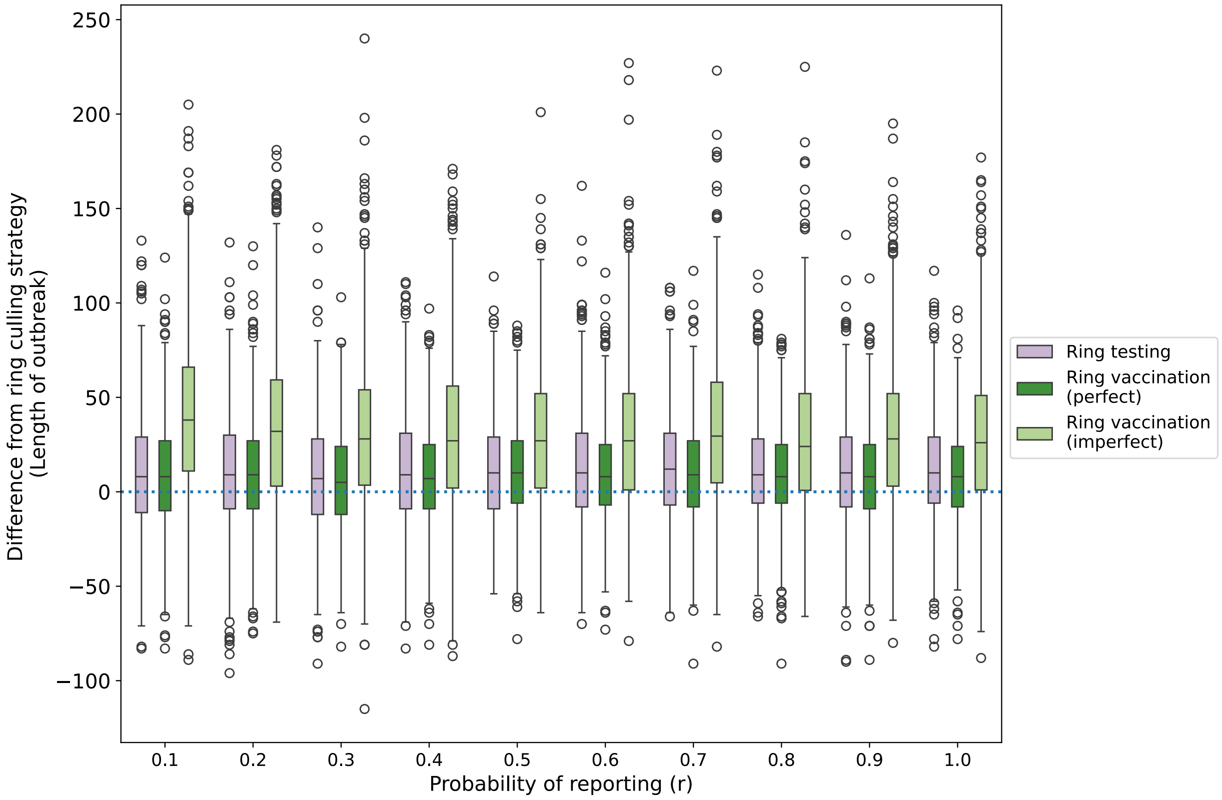

Figure 2: The difference in length of outbreak for each simulation from a ring culling strategy for ring testing and ring vaccination (perfect and imperfect) strategies. A positive value means the considered strategy is worse than ring culling, i.e. it results in a longer outbreak. A negative value means the considered strategy is better than ring culling i.e. it results in a shorter outbreak. 1000 simulations were generated per r value for each strategy. Simulations where the epidemic dies out before being detected were discarded (32 simulations).

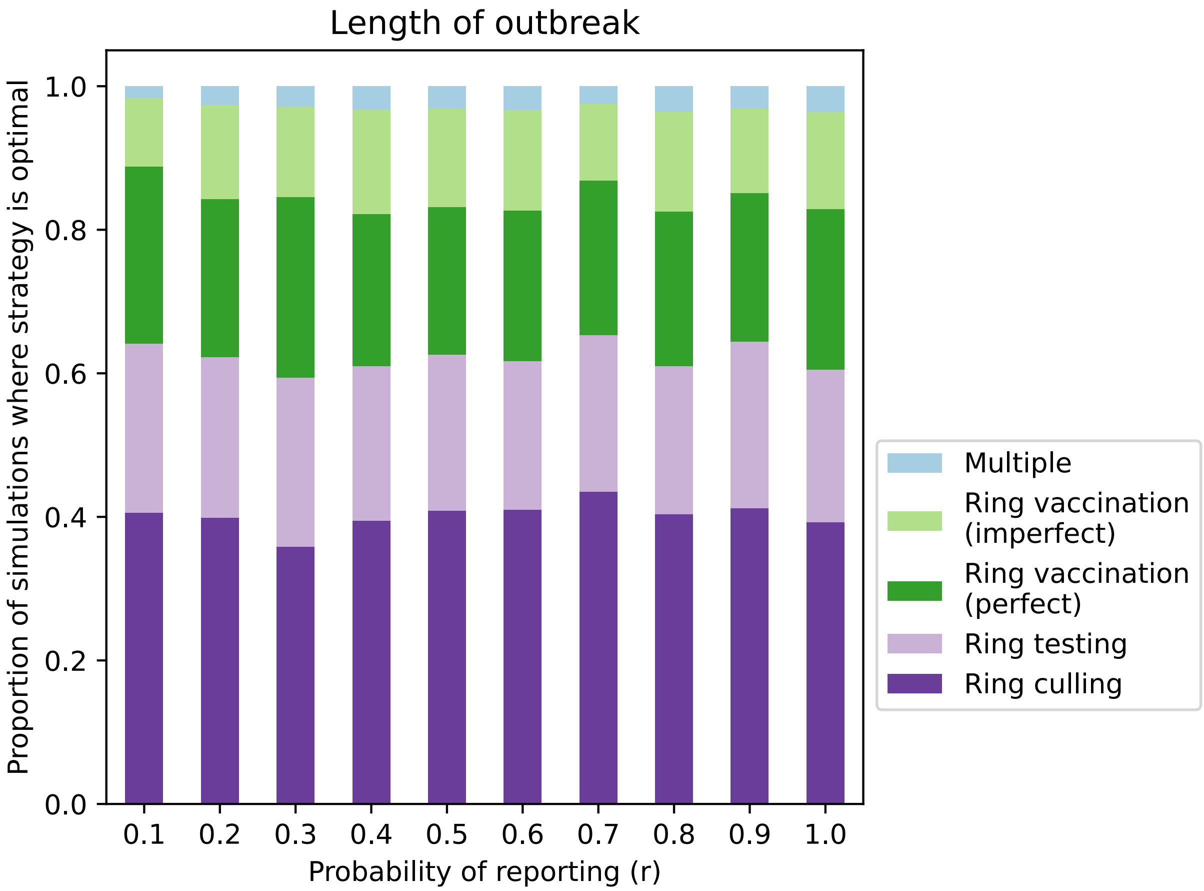

Figure 3: The proportion of simulations for which ring culling, ring testing and ring vaccination strategies are the optimal strategy to minimise the outbreak length. 1000 simulations were generated for each r value and simulations where the epidemic dies out before being detected were discarded. ‘Multiple’ indicates simulations where there was more than one optimal strategy.

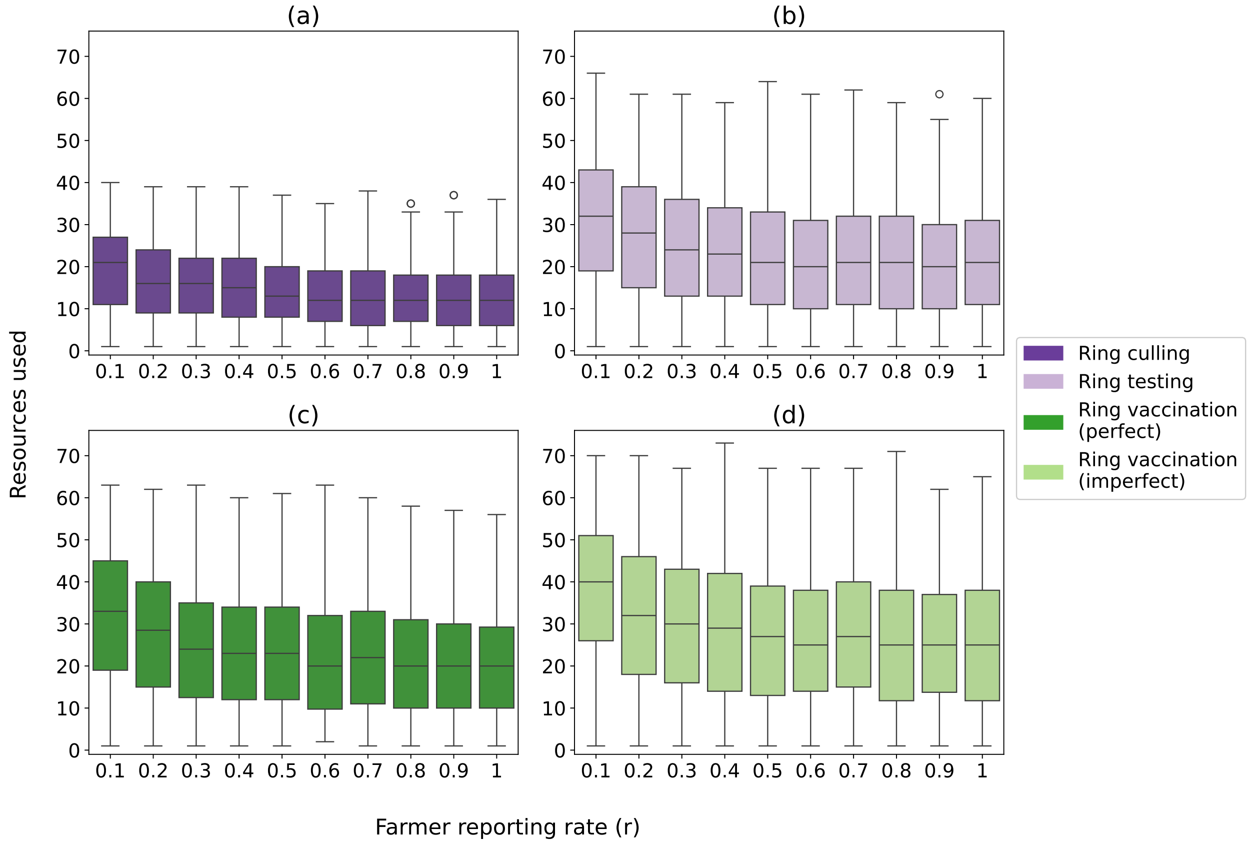

Figure 4: The total number of resources used under strategies of culling infected properties and movement restrictions combined with (a) ring culling, (b) ring testing, (c) ring vaccination (perfect) and (d) ring vaccination (imperfect) strategies as we vary the reporting rate (r). We define resources used as the number of properties culled, tested, and/or vaccinated. 1000 simulations are generated per r value for each strategy. Simulations where epidemic dies out (32 total) are removed from the results.

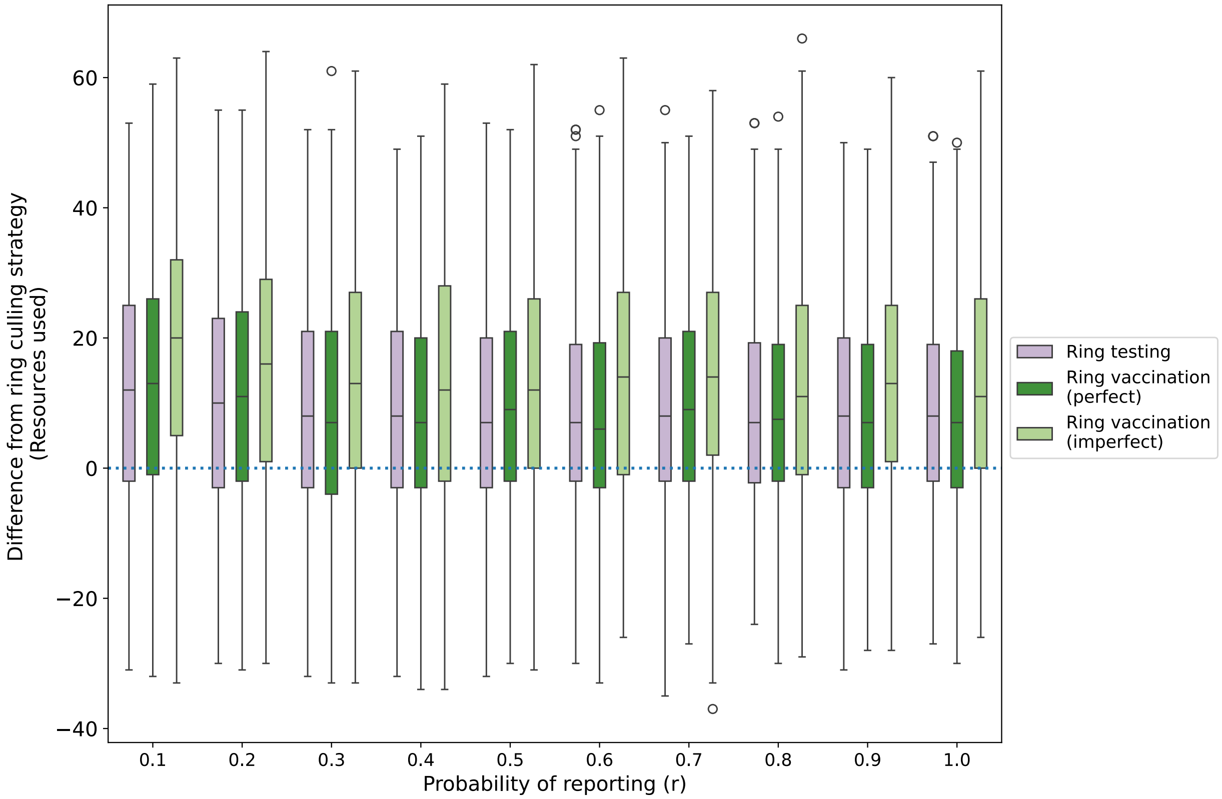

Figure 5: The difference in resources used for each simulation from a ring culling strategy for ring testing and ring vaccination (perfect and imperfect) strategies. We define resources used as the number of properties culled, tested, and/or vaccinated. A positive value means the considered strategy is worse than ring culling, i.e. it results in more resources used. A negative value means the considered strategy is better than ring culling i.e. it results in fewer resources used. 1000 simulations were generated per r value for each strategy. Simulations where the epidemic dies out before being detected were discarded (32 simulations).

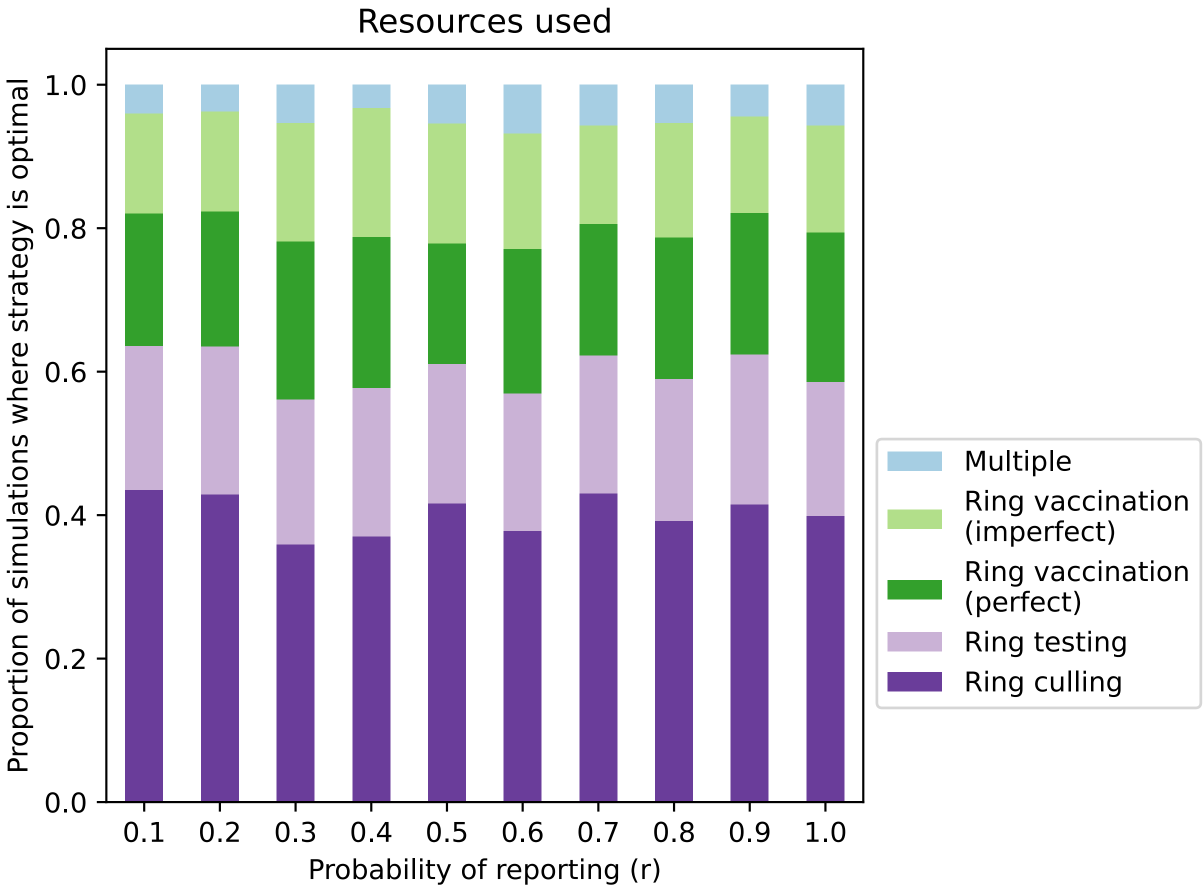

Figure 6: The proportion of simulations for which ring culling, ring testing and ring vaccination strategies are the optimal strategy to minimise total resources used. We define resources used as the number of properties culled, tested, and/or vaccinated. 1000 simulations were generated for each r value and simulations where the epidemic dies out before being detected were discarded. ‘Multiple’ indicates simulations where there was more than one optimal strategy.
